# Evaluation of Chitotriosidase and Neopterin as Biomarkers of Microvascular Complications in Patients with Type 1 Diabetes Mellitus

**DOI:** 10.3390/diagnostics11020263

**Published:** 2021-02-08

**Authors:** Ancuța Cutaș, Cristina Drugan, Gabriela Roman, Adriana Rusu, Cristina Sorina Cătană, Andrei Achimaș-Cadariu, Tudor Drugan

**Affiliations:** 1 Department of Medical Informatics and Biostatistics, “Iuliu Hațieganu” University of Medicine and Pharmacy, 400012 Cluj-Napoca, Romania; cutas.ancuta@umfcluj.ro (A.C.); aachimas@umfcluj.ro (A.A.-C.); tdrugan@umfcluj.ro (T.D.); 2 Department of Medical Biochemistry, “Iuliu Hațieganu” University of Medicine and Pharmacy, 400012 Cluj-Napoca, Romania; ccatana0128@gmail.com; 3 Department of Diabetes, Nutrition and Metabolic Diseases, “Iuliu Hațieganu” University of Medicine and Pharmacy, 400012 Cluj-Napoca, Romania; groman@umfcluj.ro (G.R.); adriana.rusu@umfcluj.ro (A.R.)

**Keywords:** diabetes mellitus, chitotriosidase, neopterin, inflammation, microvascular complications

## Abstract

The chronic complications of diabetes mellitus (DM) are accompanied by inflammatory manifestations. Our study aimed to evaluate a possible association between the inflammatory status (reflected by serum chitotriosidase and neopterin) and the timely evolution and occurrence of chronic microvascular complications in patients with type 1 DM. This observational, cross-sectional study included 82 type 1 DM patients from the Centre for Diabetes, Nutrition and Metabolic Diseases, Cluj-Napoca, Romania. Our results demonstrated a link between the extent of inflammation, evaluated by the enzymatic activity of circulating chitotriosidase, and the onset of microvascular complications, especially diabetic neuropathy and retinopathy. Chitotriosidase enzymatic activity showed an ascending evolution over time. In non-smoking patients, the increase in chitotriosidase activity was correlated with the extent of microalbuminuria and the decline of glomerular filtration rate, while in smokers, only the presence of a positive correlation between chitotriosidase activity and disease progression was noticed. According to our results, the time span between the moment of diagnosis and the onset of microvascular complications was longer in non-smokers than in smokers. These results also imply that increased chitotriosidase activity may be a predictor of endothelial dysfunction in type 1 DM.

## 1. Introduction

Diabetes mellitus (DM) is increasingly considered a major public healthcare problem. Currently, 463 million people worldwide are affected by this disease. The International Diabetes Federation (IDF) estimates that, by 2045, the number of patients with DM may reach 700 million. In Europe, the latest estimates show that 59.3 million people are affected, of whom 286.000 are children and adolescents (aged 0–19 years) with type 1 DM. In Romania, the prevalence of this disease has reached 8.8%, with approximately 1 in 11 people being diagnosed with DM [1].

Chronic complications of DM affect multiple organs and systems and are largely responsible for the morbidity and mortality associated with this disease. The risk of chronic complications is directly proportional to the increased level of blood glucose, and their onset is usually detected after 10 years of persistent hyperglycaemia [2]. The control of DM is achieved by regular monitoring of blood glycaemia and glycated haemoglobin (HbA1c), which is the result of non-enzymatic glycosylation of haemoglobin. HbA1c is widely used as an indicator of glycaemic control within 2–3 months prior to the testing. The link between HbA1c and the average blood glucose levels in previous months is well established, as is its role as an indicator of the risk of chronic complications. Its clinical utility has also been confirmed by its strong association with cardiovascular morbidity: an increase in HbA1c of only 1% may cause a rise in morbidity and mortality by cardiovascular events of up to 10% [3,4].

DM is associated with an accelerated evolution of atherosclerosis, affecting all blood vessels, but especially those of the heart, brain and extremities. Owing to its specific pathophysiology, diabetic retinopathy is the first cause of blindness in patients aged 20–74 years. Likewise, the first cause of chronic kidney disease is diabetic nephropathy [5].

DM develops as a result of a combination between insulin resistance and pancreatic beta (β) cell failure, which are aggravated by oxidative stress, endoplasmic reticulum stress, lipotoxicity and glucotoxicity. These cellular alterations induce an inflammatory response that plays an active role in the pathophysiology of DM. In response to environmental stress, macrophages, endothelial cells and adipocytes release inflammatory cytokines. Some of them are represented by tumour necrosis factor alpha (TNF-α), interleukin 1 (IL-1), interleukin 6 (IL-6) and chemokines, such as monocyte chemoattractant protein-1 (MCP-1) and interleukin 8 (IL-8). Their combined effects trigger the release of acute phase markers (e.g., C-reactive protein) and the accumulation of pancreatic amyloid [6]. Circulating inflammatory markers induce a chronic state of low-grade inflammation, which leads to an increased risk of vascular complications [7].

Two markers of macrophage activation, namely, chitotriosidase and neopterin, play an important part in the activation of immunological and inflammatory response. Currently, their activity is still the focus of intensive debate, as the exact mechanism by which they regulate these functions is poorly understood, and there is controversy regarding their protective or harmful effects [8].

Among chitinases, human chitotriosidase is the best characterized enzyme, both from a clinical and biological point of view. Proinflammatory cytokines, such as granulocyte macrophage colony stimulating factor (GM-CSF),TNF-α, lipopolysaccharides (LPS), bacterial muramyl dipeptide and prolactin stimulate the expression of chitotriosidase in monocyte-derived macrophages, while interferon gamma (IFN-γ) and IL-4 inhibit the expression of chitotriosidase [7,9,10].

Elevated serum levels have been described in many pathologies involving immune system dysfunctions [7,9]. Besides being an important marker in lysosomal storage disorders, such as Gaucher and Niemann–Pick disease, increased enzymatic activity of chitotriosidase is characteristic of atherosclerosis, non-alcoholic hepatic steatosis and juvenile arthritis. Under normal and inflammatory conditions, chitotriosidase is mainly secreted by circulating polymorphonuclear neutrophils and activated macrophages, respectively. An overexpression of its corresponding gene, called chitinase 1 (CHIT1) could result in the initiation and persistence of chronic inflammation in patients with DM [11,12].

Neopterin belongs to the class of pteridines and is derived from guanosine triphosphate (GTP). It is increasingly recognized as an independent marker of immune activation and an important predictive marker of cardiovascular risk. Human monocytes or macrophages appear to be the most relevant source of neopterin and 7,8-dihydrononeopterin. Cytokines, such as IL-2, TNF-α, LPS, GM-CSF, human immunodeficiency virus (HIV) and other viruses, may stimulate the release of interferon gamma (IFN-γ) from T cells and, thus, trigger the release of neopterin [13]. Elevated concentrations have been detected in patients with viral and parasitic infections, allograft rejections and autoimmune diseases. Several studies have correlated the level of circulating neopterin with the activity of systemic lupus erythematosus, Wegener′s granulomatosis, dermatomyositis, Crohn′s disease and ulcerative rectocolitis [14]. Moreover, neopterin levels allow the discrimination between rheumatoid arthritis and osteoarthritis. Furthermore, increased concentrations of serum neopterin have been described in malignant and neurodegenerative diseases, in patients with Gaucher disease (characterised by an interaction between activated macrophages and chronic immune stimulation) and in DM patients, during the evolution of chronic complications [13,14,15].

Our study aimed to evaluate a possible association between the inflammatory status, reflected by circulating chitotriosidase and neopterin levels and the occurrence of chronic microvascular complications in patients with type 1 DM. We also sought to investigate any possible relationship between the studied biomarkers and the length of disease evolution until the occurrence of chronic complications.

## 2. Materials and Methods

### 2.1. Patients

This observational cross-sectional study included 82 patients with type 1 DM, consecutively selected from the Centre for Diabetes, Nutrition and Metabolic Diseases, Cluj-Napoca, Romania, between January 2018 and November 2019. The diagnosis of DM and its chronic complications were established according to the criteria of the American Diabetes Association [3]. Patients with acute infections (urinary, respiratory, digestive, ulcerative infections), liver damage (alanine aminotransferase activity more than 3 times the upper physiological limit), malignancies and haematological disorders, patients with an estimated glomerular filtration rate below 30 mL/min/1.73 m² and those undergoing anti-inflammatory treatment for any other reasons were excluded. All these medical conditions could interfere with the level of chitotriosidase, neopterin and HbA1c [8,14,16]. All patients were treated with personalised doses of long- and short-acting insulin and some of them used insulin pumps.

The research protocol was approved by the Ethics Committee of the University of Medicine and Pharmacy Cluj-Napoca, Romania (no. 408/08.11.2017), in accordance with the revised Helsinki Declaration of 2000. Before enrolment, all selected patients signed an informed written consent form.

### 2.2. Study Protocol

Data collection was performed using patient medical records, for the following items: age and gender, height, abdominal circumference, weight and body mass index (BMI), time span since the diagnosis of DM, fasting glycaemia and HbA1c values, complete blood count indices and smoking status. We also recorded the eventual presence and type of chronic microvascular complications (retinopathy, neuropathy, microalbuminuria), the coexistence of arterial hypertension and of any other associated chronic disorders. Serum chitotriosidase enzymatic activity was measured by a fluorometric method, using an artificial substrate [17] (reference range 3–100 nmol/h/mL) and circulating neopterin levels were detected by an enzyme-linked immunosorbent (ELISA) assay, according to the manufacturer′s instructions (Human Neopterin ELISA Kit, Wuhan Fine Biotech, China).

Chronic microvascular complications of DM were recorded as present or absent, according to the eventual detection, or complete absence, of clinical signs or symptoms of neuropathy [18] and retinopathy (diagnosed using the Early Treatment Diabetic Retinopathy Study (ETDRS) classification) [19]. Microalbuminuria was divided into three stages, based on urine albumin to creatinine ratio (UACR): stage A1—below 30 mg/g, stage A2—between 30 and 300 mg/g and stage A3—over 300 mg/g, according to the Kidney Disease Improving Global Outcomes (KDIGO) classification [20]. The coexistence of other autoimmune or inflammatory diseases (such as thyroiditis, celiac disease, allergic asthma, psoriasis, Buerger′s disease, neuropathic ulcer, vitiligo, rheumatoid arthritis, Basedow′s disease, chronic obstructive pulmonary disease) was taken into account in data analysis. Hypercholesterolemia was recorded if total cholesterol level exceeded 200 mg/dL, hypertriglyceridemia was defined as serum triglyceride levels above 150 mg/dL and low-density lipoprotein cholesterol (LDL cholesterol) values were calculated according to the Friedewald Equation [21].

### 2.3. Statistical Analysis

Statistical analysis was performed with IBM SPSS Statistics for Windows, Version 25.0. Armonk, NY: IBM Corp. The normality of distribution of quantitative variables was tested using the Kolmogorov–Smirnov test. Non-parametric (Mann–Whitney U and Kruskal–Wallis) tests were subsequently used for data analysis. Because of the highly dispersed values of quantitative variables that did not display a normal distribution, we used the Spearman correlation coefficient and its significance test. Quantitative variables were presented as means and standard deviations, or as medians and quartiles. For the description of qualitative variables, we used frequency tables and employed contingency tables to analyse their potential associations. For a better understanding of our approach, we chose to present the mean and standard deviation, even for variables that were not normally distributed.

## 3. Results

### 3.1. Description of Clinical and Biological Parameters

Our study comprised 82 patients with type 1 DM, of whom 44 (53.66%) were men. Neuropathy was present in 58.54% and retinopathy in 60.98% of patients. Microalbuminuria was present as follows: 75.61% had stage A1, 19.51% stage A2 and 4.88% stage A3. Only seven (15.9%) male and eight (21.05%) female patients received treatment with insulin by continuous subcutaneous infusion (insulin pump), the others being treated with insulin in basal-bolus regimen.

The mean age of the patients was 44 years (±13.39), with a body mass index (BMI) of 25.65 kg/m² (±4.37). The mean value of HbA1c was 8.85% (±1.78), and the mean duration of diabetes was 20 years (Q1 = 13; Q3 = 31). The mean glomerular filtration rate was 97.27 mL/min/1.73 m² (±20.68), the median UACR was 10.86 mg/g (Q1 = 5.03; Q3 = 28.68), the mean fasting glycaemia at diagnosis was 173 mg/dL (Q1 = 124; Q3 = 219), the mean insulin dose per kilogram body weight was 0.71 IU/kg (±0.26), and the mean value of LDL cholesterol was 107.49 mg/dL (±33.33). The median value of serum chitotriosidase activity was 147.5 nmol/mL/h (Q1 = 80; Q2 = 240), and that of neopterin was 9.31 ng/mL (Q1 = 6.18; Q2 = 16.03). Full blood count showed a mean haemoglobin value of 14.27 g/dL (±1.8), a number of 262.4 × 10^9^ platelets/L (±61.94) and a number of 7.03 × 10^9^ leukocytes/L (±2.13). Smoking status was found in 12 (14.63%) male patients and in 10 (12.20%) female patients. Among the associated pathologies, chronic inflammatory diseases were present in 23.17% of patients.

No significant differences in age, disease duration, HbA1c, BMI, serum chitotriosidase or neopterin levels were noticed, according to neither gender distribution (Table 1) nor microvascular complications occurrence (Table 2).

### 3.2. Overall Differences in Chitotriosidase and Neopterin According to Microvascular Complications

For the whole patient cohort, we found statistically significant differences between the enzymatic activity of chitotriosidase in patients with neuropathy and retinopathy, compared to those without these complications. However, no significant differences in chitotriosidase activity were observed among patients with microalbuminuria, regardless of its evolutionary stage. For the serum level of neopterin, no statistically significant results were obtained for any of the existing microvascular complications (Table 3).

### 3.3. Confounding Factors: Smoking and Associated Inflammatory Diseases

For the entire patient cohort, a statistically significant difference between the enzymatic activity of chitotriosidase in smoking and non-smoking subjects was noticed. In the case of neopterin, the statistical significance was not maintained. From a statistical point of view, coexisting inflammatory diseases did not influence the values of studied biomarkers (Table 4).

### 3.4. Differences in Chitotriosidase and Neopterin According to Microvascular Complications in Patients with Confounding Factors

Within the subgroup of patients with confounding factors, no statistically significant differences in chitotriosidase activity or neopterin levels were observed between the subjects with different types of chronic complications and those without complications (Table 5).

### 3.5. Differences in Chitotriosidase and Neopterin According to Microvascular Complications in Patients without Confounding Factors

The enzymatic activity of chitotriosidase in non-smoking patients has reached the threshold of statistical significance for neuropathy and retinopathy, but not for microalbuminuria. For neopterin, no statistically significant differences were observed for any of the chronic complications (Table 6). When comparing the two biomarkers in patients with neuropathy, the diagnostic accuracy of chitotriosidase was superior to that of neopterin, with an area under the curve (AUC) of 0.843 and an associated confidence interval between 0.727–0.959 (*p* < 0.001).

### 3.6. Correlation of Chitotriosidase and Neopterin with DM Duration and HbA1c Levels

Of the two biomarkers of macrophage activation, only chitotriosidase was positively correlated with the duration of DM. Neither of the two biomarkers reached statistical significance in terms of correlation with HbA1c or with the therapeutic insulin dose (Table 7).

### 3.7. Stratified Analysis of the Correlations between Chitotriosidase and the Parameters of DM Evolution According to Smoking Exposure

A moderate, positive, statistically significant correlation was observed in both smoking and non-smoking patients between chitotriosidase enzymatic activity and disease duration. In non-smokers, both the age of the patients and the UACR were positively and significantly correlated with the inflammatory biomarker. Another correlation that reached statistical significance in non-smokers was a negative one, between chitotriosidase activity and the glomerular filtration rate (GFR) (Table 8).

## 4. Discussion

Our study aimed to evaluate the association between chronic inflammation and microvascular complications in patients with type 1 DM. Given that, on average, our patients had an increased percentage of HbA1c (8.85%), a long-term evolution of DM (20 years) and were mostly overweight (25.65 kg/m^2^) [5], microvascular complications were distributed as follows: 58.54% had neuropathy, 60.98% were affected with retinopathy and 75.61% with microalbuminuria. Smoking patients represented 26.83% of the whole group, and those with other chronic inflammatory diseases accounted for 23.17%.

Our results demonstrated an increase in chitotriosidase activity in patients with microvascular complications, compared to those without complications, with statistically significant differences for neuropathy and retinopathy. High levels of circulating chitotriosidase were also associated with long-term evolution of DM, microalbuminuria and an important decline in GFR.

As biomarkers of macrophage activation, both chitotriosidase and chitinase-3-like protein 1 (CHI3L1), also called YKL-40, have been studied mainly in patients with type 2 DM. Previous studies have shown that in both type 1 and 2 DM, chitotriosidase activity was correlated with age, GFR and UACR, but not with glycaemia or HbA1c [22,23,24,25].

Among chronic microvascular complications, neuropathy is the most common. Due to the lack of specific symptoms, its diagnosis can be easily overlooked, especially in the early stages of evolution. Our study has shown a considerable increase in serum chitotriosidase activity in patients with neuropathy, compared to those without it. The difference became even more important if neuropathy was associated with long-term disease duration (average time length of 22 years, with a lower limit of 10 years). These results are consistent with those reported in patients with type 2 DM, affected by this complication [25].

For the evaluation of circulating neopterin, we considered the reference range suggested by Nazar et al. [26]. Despite increased levels, the variation of neopterin did not reach statistical significance in patients with neuropathy, contrary to the results obtained by Elbarbary et al. [27], who demonstrated a link between neopterin levels and nerve conduction latency (both sensory and motor), in paediatric patients with type 1 DM.

After 20 years of evolution, most DM patients have some degree of retinal damage, which is the most common cause of blindness [4]. In this study, patients with retinopathy (regardless of its progression) had significantly higher values of serum chitotriosidase, than those in whom retinopathy was still undetected. Our results support those observed in patients with type 2 DM and retinopathy, where the protein YKL-40, a member of the same family as chitotriosidase, had been proposed as a predictive marker of macular oedema and retinal detachment [7,25,28,29]. As in the case of neuropathy, serum levels of neopterin were increased in patients with retinopathy, but this variation lacked statistical significance.

There is clear evidence that the pathogenesis of diabetic nephropathy is multifactorial, but inflammation plays a key pathophysiological role [5]. After 10–15 years of evolution, around 20–30% of diabetic patients develop the early stages of this complication. In the case of diabetic nephropathy, unlike for other microvascular complications, genetic predisposition plays an important role, explaining the increased susceptibility of some patients to the occurrence of renal complications [26,30]. An increased rate of albuminuria reflects vascular damage, as part of renal endothelial dysfunction [7]. However, in our patients, serum chitotriosidase values in different stages of microalbuminuria did not display statistically significant differences. Chitotriosidase values showed an ascending variation in the first two stages of nephropathy, yet in the third stage, they decreased, most likely due to the occurrence of fibrosis. During the entire length of disease evolution, circulating chitotriosidase increased progressively, a tendency that was positively correlated with UACR and negatively correlated with GFR. Similarly, the relationship between elevated serum chitotriosidase (and also YKL-40) and the occurrence of albuminuria has already been reported in patients with type 2 DM [24,25,31,32]. Again, neopterin variation did not reach statistical significance among our patients with diabetic nephropathy, although there is evidence of a relationship between elevated neopterin levels and the progression of kidney disease in patients with type 1 DM [33].

Because 26.83% of our patients were smokers and 23.17% had other chronic inflammatory diseases, we investigated these two variables as potential confounders. Chitotriosidase activity was undoubtedly higher in smokers, compared to non-smokers, with a statistically significant difference between the two patient subgroups. We presume that this result may be explained by the persistent inflammatory response generated by smoking [34,35].

According to our results, smoking and coexisting inflammatory diseases could mask the evolution of DM complications. Therefore, in these patients, no statistically significant differences were observed between the levels of inflammatory markers in subjects with and without chronic complications, although chitotriosidase had higher values in association with neuropathy. In the absence of confounding factors, significant differences were obvious for neuropathy and retinopathy, but not for microalbuminuria.

Our study showed a statistically significant correlation between chitotriosidase values and the duration of the disease for all patients and in both subgroups (smokers and non-smokers). We only found a correlation between chitotriosidase activity and age in non-smokers, while in smokers it disappeared, due to the smoking-induced activation of the enzyme. Unlike the Ramanathan study [36], which showed a correlation between chitotriosidase levels and increasing age, in our research, the average patient age was much lower, as we did not include older patients with multiple age-induced inflammatory conditions. Therefore, we suppose the increase in chitotriosidase levels over the course of disease may be a direct consequence of disease evolution and not an age-induced phenomenon.

## 5. Conclusions

To the best of our knowledge, this is the first evaluation of chitotriosidase as an inflammatory marker in patients with type 1 DM and microvascular complications. We demonstrated a link between the extent of inflammatory status, quantified by the enzymatic activity of chitotriosidase, and the occurrence of neuropathy and retinopathy in type 1 DM. Circulating chitotriosidase showed an increased progression over time. In non-smoking patients, its evolution was correlated with the aggravation of microalbuminuria and the decline of GFR, while in smokers, only a positive correlation between chitotriosidase activity and disease progression was observed. Moreover, according to our results, the length of disease evolution between the moment of diagnosis and the occurrence of microvascular complications was longer in non-smokers than in smokers, highlighting the impact of chronic inflammation on the progression of DM. In our study, circulating levels of neopterin were not associated with any of the microvascular complications in patients with type 1 diabetes.

## Figures and Tables

**Table 1:** Distribution of variables according to the gender of patients with type 1 diabetes.

	Male	Female	*p*-Value
	Mean (±SD)	Mean (±SD)	
Age (years)	43.34 (±13.46)	45.57 (±13.39)	0.454 *
Disease duration (years)	21.22 (±12.51)	23.71 (±11.53)	0.272 **
HbA1c (%)	8.97 (±2.00)	8.70 (±1.51)	0.507 *
Insulin dose (UI/kg)	0.76 (±0.30)	0.63 (0.19)	0.018 *
BMI (kg/m²)	25.67 (±4.14)	25.62 (±4.67)	0.965 *
Chitotriosidase (nmol/mL/h)	213.04 (±179.17)	168.91 (±136.82)	0.352 **
Neopterin (ng/mL)	12.49 (±6.98)	10.90 (±8.87)	0.096 **

* T-Test for equal variances. ** Mann–Whitney U-test for independent samples. HbA1c (%)—glycated haemoglobin, BMI—body mass index, IU—international units, kg—kilogram.

**Table 2:** Microvascular complications in patients with type 1 diabetes.

		Type 1 Diabetes Mellitus			*p*-Value
		Male	Female	Total	
Neuropathy	absent	18 (21.95%)	16 (19.51%)	34	0.913 *
	present	26 (31.71%)	22 (26.83%)	48	
Retinopathy	absent	16 (19.51%)	16 (19.51%)	32	0.595 *
	present	28 (34.15%)	22 (26.83%)	50	
Microalbuminuria	A1	30 (36.59%)	32 (39.02%)	62	0.098 *
	A2	10 (12.20%)	6 (7.32%)	16	
	A3	4 (4.88%)	0 (0.00%)	4	
TOTAL	-	44	38	82	-

* Chi-square uncorrected. A1, A2, A3—stages of microalbuminuria, based on urine albumin to creatinine ratio (UACR).

**Table 3:** Mean values of the studied biomarkers according to the type of chronic complications.

		Chitotriosidase (nmol/mL/h)	*p*-Value	Neopterin (ng/mL)	*p*-Value
		Mean (±SD)		Mean (±SD)	
Neuropathy	absent	112.41 (±93.39)	0.000 *	12.01 (±7.23)	0.645 *
	present	244.68 (±174.92)		11.58 (±8.41)	
Retinopathy	absent	137 (±129.09)	0.003 *	13.45 (±9.17)	0.157 *
	present	226.56 (±170.38)		10.67 (±6.85)	
Microalbuminuria	A1	177.58 (±153.96)	0.132 **	11.94 (±8.22)	0.720 **
	A2	245.62 (±193.60)		10.71 (±7.02)	
	A3	188.75 (±90.03)		13.13 (±7.58)	

* Mann–Whitney U-test for independent samples. ** Kruskal–Wallis test for independent samples. A1, A2, A3—stages of microalbuminuria, based on urine albumin to creatinine ratio (UACR).

**Table 4:** Influence of smoking and associated inflammatory diseases as confounding factors in patients with type 1 diabetes.

	Chitotriosidase (nmol/mL/h)	*p*-Value	Neopterin (ng/mL)	*p*-Value
	Mean (±SD)		Mean (±SD)	
Non-smoking patients	169.64 (±138.92)	0.023 *	11.70 (±7.33)	0.921 *
Smoking patients	253.09 (±201.03)		11.91 (±9.48)	
No associated inflammatory diseases	173.75 (±132.77)	0.238	12.33 (±8.28)	0.416
Associated inflammatory diseases	253.33 (±225.97)		9.85 (±6.32)	

* Mann–Whitney U-test for independent samples.

**Table 5:** Mean values of the studied biomarkers in patients with confounding factors, according to microvascular complications.

		Smoking Patients				Patients with Associated Inflammatory Diseases			
		Chitotriosidase (nmol/mL/h)		Neopterin (ng/mL)		Chitotriosidase (nmol/mL/h)		Neopterin (ng/mL)	
		Mean (±SD)	*p*-Value	Mean (±SD)	*p*-Value	Mean (±SD)	*p*-Value	Mean (±SD)	*p*-Value
Neuropathy	absent	126.66 (50.33)	0.185 *	8.36 (5.01)	0.484 *	177.50 (172.62)	0.291 *	12.41 (9.22)	0.711 *
	present	274.16 (209.66)		12.70 (10.15)		291.25 (246.31)		8.36 (3.52)	
Retinopathy	absent	264 (270.51)	0.548 *	16.67 (15.77)	0.446 *	135 (94.73)	0.056 *	11.94 (8.67)	0.778 *
	present	249.68 (185.26)		10.51 (6.84)		328.63 (255.70)		8.33 (3.64)	
Micro-Albuminuria	A1	244.23 (184.27)	0.961 **	11.75 (10.55)	0.835 **	196.78 (176.44)	0.233 **	10.68 (±6.83)	0.468 **
	A2	298.33 (273.81)		12.78 (8.93)		498.33 (343.01)		6.19 (2.50)	
	A3	175.00 (21.21)		10.45 (5.89)		310		8.43	

* Mann–Whitney U-test for independent samples. ** Kruskal–Wallis test for independent samples. A1, A2, A3—stages of microalbuminuria, based on urine albumin to creatinine ratio (UACR).

**Table 6:** Mean values of the studied biomarkers in patients without confounding factors, according to microvascular complications.

		Non-Smoking Patients				No Associated Inflammatory Diseases			
		Chitotriosidase (nmol/mL/h)		Neopterin (ng/mL)		Chitotriosidase (nmol/mL/h)		Neopterin (ng/mL)	
		Mean (±SD)	*p*-Value	Mean (±SD)	*p*-Value	Mean (±SD)	*p*-Value	Mean (±SD)	*p*-Value
Neuropathy	absent	110.89 (97.36)	0.000 *	12.49 (7.40)	0.325 *	96.80 (58.16)	0.000 *	11.90 (6.83)	0.967 *
	present	226.37 (150.57)		10.90 (7.28)		228.71 (144.01)		12.65 (9.30)	
Retinopathy	absent	111.60 (62.52)	0.016 *	12.85 (7.73)	0.183 *	139.71 (13.95)	0.027 *	13.95 (9.46)	0.218 *
	present	215 (164.30)		10.75 (6.96)		196.21 (124.94)		11.33 (7.41)	
Micro-Albuminuria	A1	158.33 (140.56)	0.171 **	11.99 (7.54)	0.361 **	171.47 (147.83)	0.364 **	12.34 (8.64)	0.791 **
	A2	214 (133.62)		9.46 (5.76)		187.30 (87.14)		11.75 (7.37)	
	A3	202.5 (152.02)		15.81 (10.43)		148.33 (48.56)		14.70 (8.45)	

* Mann–Whitney U-test for independent samples. ** Kruskal–Wallis test for independent samples. Legend: A1, A2, A3—stages of microalbuminuria, based on urine albumin to creatinine ratio (UACR).

**Table 7:** Correlation of inflammatory markers with the timely evolution of diabetes mellitus.

	Disease Duration (Years)		HbA1c (%)		Insulin Dose IU/kg	
	Spearman Correlation Coefficient	*p*-Value	Spearman Correlation Coefficient	*p*-Value	Spearman Correlation Coefficient	*p*-Value
Chitotriosidase (nmol/mL/h)	0.597	0.000 *	−0.055	0.632 *	0.048	0.679 *
Neopterin (ng/mL)	−0.103	0.358 *	−0.049	0.660 *	−0.163	0.146 *

* Spearman′s rho correlation is significant at the 0.01 level (2-tailed). HbA1c—glycated haemoglobin, IU—international units, kg—kilogram.

**Table 8:** Correlation between chitotriosidase activity and the parameters of disease evolution in patients with type 1 diabetes.

	Chitotriosidase (nmol/mL/h)			
	Non-Smokers		Smokers	
	Spearman Correlation Coefficient	*p*-Value	Spearman Correlation Coefficient	*p*-Value
Age (years)	0.747	0.000 *	0.087	0.707
Disease duration (years)	0.623	0.000 *	0.654	0.001 *
Glycemia (mg/dL)	0.073	0.590	−0.292	0.198
HbA1c (%)	−0.108	0.425 *	−0.237	0.302 *
GFR (mL/min/1.73 m^2^)	−0.577	0.000 *	−0.385	0.085
UACR (mg/g)	0.329	0.013 *	−0.028	0.904 *
Low density lipoprotein cholesterol (mg/dL)	−0.073	0.591 *	−0.218	0.343

* Spearman′s rho correlation is significant at the 0.01 level (2-tailed). HbA1c—glycated haemoglobin, GFR—glomerular filtration rate, UACR—urine albumin to creatinine ratio.

## Data Availability

Due to the fact that data sharing is not in accordance with the consent provided by participants on the use of their confidential data, authors cannot make study data available in a publicly accessible repository.
